# Can Differences in Host Behavior Drive Patterns of Disease Prevalence in Tadpoles?

**DOI:** 10.1371/journal.pone.0024991

**Published:** 2011-09-15

**Authors:** Matthew D. Venesky, Jacob L. Kerby, Andrew Storfer, Matthew J. Parris

**Affiliations:** 1 Department of Biological Sciences, University of Memphis, Memphis, Tennessee, United States of America; 2 Department of Integrative Biology, University of South Florida, Tampa, Florida, United States of America; 3 Biology Department, University of South Dakota, Vermillion, South Dakota, United States of America; 4 School of Biological Sciences, Washington State University, Pullman, Washington, United States of America; Institute of Marine Research, Norway

## Abstract

Differences in host behavior and resistance to disease can influence the outcome of host-pathogen interactions. We capitalized on the variation in aggregation behavior of Fowler's toads (*Anaxyrus* [ = *Bufo*] *fowleri*) and grey treefrogs (*Hyla versicolor*) tadpoles and tested for differences in transmission of *Batrachochytrium dendrobatidis* (*Bd*) and host-specific fitness consequences (i.e., life history traits that imply fitness) of infection in single-species amphibian mesocosms. On average, *A. fowleri* mesocosms supported higher *Bd* prevalences and infection intensities relative to *H. versicolor* mesocosms. Higher *Bd* prevalence in *A. fowleri* mesocosms may result, in part, from higher intraspecific transmission due to the aggregation of tadpoles raised in *Bd* treatments. We also found that, independent of species, tadpoles raised in the presence of *Bd* were smaller and less developed than tadpoles raised in disease-free conditions. Our results indicate that aggregation behavior might increase *Bd* prevalence and that *A. fowleri* tadpoles carry heavier infections relative to *H. versicolor* tadpoles. However, our results demonstrate that *Bd* appears to negatively impact larval growth and developmental rates of *A. fowleri* and *H. versicolor* similarly, even in the absence of high *Bd* prevalence.

## Introduction

Traditionally, ecologists have focused on the effects of disturbance, competition, and predation when studying species interactions [1], [2]. However, recent studies underscore the impacts that parasites and pathogens (hereafter “pathogens”) have on communities [3], [4], [5]. For example, pathogens can directly affect host populations by causing mortality [6] or indirectly by altering life history traits, such as growth and developmental rates [7]. Additionally, sub-lethal infections can alter normal host behaviors such as feeding [8], antipredator [9], and thermoregulatory [10] behavior, each of which can shape the ecological interactions within a community. Indeed, certain behaviors by non-infected hosts can increase their chances of becoming infected. For example, hosts with promiscuous mating strategies may increase the probability of contact with an infected host [11]. Since pathogen transmission is one of the driving forces behind pathogen regulation of host populations [12], identifying how pathogen-induced changes in host behavior affect pathogen transmission is a central component of host–pathogen ecology.

Determining the role that pathogens play in amphibian populations is especially important given their worldwide declines [13], [14]. Chytridiomycosis, an infectious disease of amphibians, is caused by the pathogenic fungus *Batrachochytrium dendrobatidis* (*Bd*) and has been implicated as a causal agent in amphibian population declines over the past three decades [13], [15]. *Bd* is spread through aquatic environments by free-swimming zoospores [16], [17]. In tadpoles, *Bd* infections are restricted to within, or around, the keratinized labial teeth and jaw sheaths. *Bd* does not generally cause mortality in anuran tadpoles (but see [18]); however, *Bd* infections can reduce larval feeding efficiency [19], [20] and growth and developmental rates [21], [22], [23], [24].

Given the potential impacts that interspecific differences in host behavior and life history traits have on disease dynamics in host populations, understanding the effects of *Bd* on amphibians with different behaviors and life histories are a key component in understanding the ecology of this host-pathogen system. However, host variation in susceptibility and pathogen-induced life history responses are poorly understood (but see [18], [19], [23], [25]). For example, *Bd* infection reduced the foraging activity of *A. fowleri* tadpoles but not *H. chrysoscelis* tadpoles [19]. Additionally, few data exist on how far zoospores can travel and infect a susceptible host. If *Bd* transmission is limited by how far zoospores can travel, species-specific life history, or behavioral, strategies may differentially impact certain species. For example, species that aggregate may have an increased potential of disease transmission [26]. Indeed, recent evidence suggests that tadpoles of species that are highly social increase their aggregative behavior when infected with *Bd* [27].

In our study, we examined the effects of *Bd* on two species of anuran tadpoles—Fowler's toads (*Anaxyrus* [ = *Bufo*] *fowleri*) and grey treefrogs (*Hyla versicolor*)—in replicated, single-species, outdoor experimental mesocosms. These two species are ideal focal taxa because they are sympatric in ponds yet differ in their aggregation behavior and may have different patterns of pathogen transmission. Generally, *A. fowleri* tadpoles aggregate on the substrate of ponds [28], whereas *H. versicolor* tadpoles typically occur singly in the middle of the water column [29]. First, we measured the proportion of tadpoles aggregating and tested for species differences in tadpole aggregation when raised in the presence and absence of *Bd*. We then tested for species differences in *Bd* prevalence and infection intensity of tadpoles raised in the presence of *Bd*. We predicted that aggregation behavior in *A. fowleri* would facilitate higher *Bd* transmission and would result in higher *Bd* prevalence relative to *H. versicolor* mesocosms. Lastly, we tested whether raising tadpoles in the presence of *Bd* affected their rates of growth and development. We predicted that tadpoles raised in the presence of *Bd* would have reduced rates of growth and development relative to tadpoles raised in the absence of *Bd*.

## Materials and Methods

### Animal Collection and Husbandry

All tadpoles used in our experiments were derived from field-collected egg masses of *A. fowleri* and *H. versicolor* from within the Meeman Shelby State Park in Tennessee, USA (Shelby County, TN, USA) in 2009. For each species, all of the egg masses appeared to be oviposited the night before collection. Immediately after collection, eggs were transported to the laboratory at The University of Memphis. Upon hatching, tadpoles were maintained in 37.85 L glass aquaria (filled with approximately 20 L of aged tap water that was continually aerated) containers until they had reached the free-swimming stage (stage 25 [30]). We haphazardly selected a subset of tadpoles from all the clutches and combined the tadpoles from the different clutches to evenly distribute potential genetic effects on the larval traits we measured. We then randomly selected a subset out of the remaining stock of tadpoles to expose to *Bd*, which served as “pathogen source” tadpoles (see below). We kept the remainder of the tadpoles in the laboratory for 7 days before placing them in the mesocosms. Tadpoles were maintained on a 12 h light∶12 h dark photoperiod at 19°C (±1°C) and were fed a 50∶50 mixture of ground rabbit chow and Sera Micron® (a powered commercial algal-based food containing *Spirulina* and sea algae meal) *ad libitum* daily.

### *Batrachochytrium dendrobatidis* inoculation

*Bd* was grown in the laboratory on tryptone-gelatin hydrolysate-lactose (TGhL) agar in 9 cm Petri dishes according to standard protocol [16]. We harvested *Bd* zoospores (FMB 001, isolated in 2008 from an infected anuran in Shelby County, TN, USA) by adding 10.0-mL of sterile water to the cultures and collected the zoospores that emerged from the zoosporangia after 30 minutes. We exposed a subset of the laboratory born tadpoles (N = 38 per species) to an infectious dose of *Bd* by placing individual tadpoles in 50 mL water baths containing infectious concentrations of fungal zoospores (120,000 zoospores/mL) for 48 hours. Our design simulated transmission by water, one of the possible modes of *Bd* transmission in natural environments [17]. After the exposure period, the majority of the tadpoles (pathogen-source tadpoles; see below) were held by species in 37.85 L glass aquaria (filled with approximately 20 L of aged tap water that was continually aerated) in the laboratory. The pathogen source tadpoles (N = 24 per species) were held in the laboratory for 7 days before placing them in outdoor experimental tanks to allow *Bd* infections to develop (see below). The remaining tadpoles (N = 14 per species) were held individually in the laboratory for 7 days to confirm our infection protocol.

### Experimental Design

We manipulated the presence of *Bd* in replicated experimental mesocosms. We reared tadpoles in 32 polyethylene tanks (1.83 m diameter) positioned in an array at the University of Memphis Edward J. Meeman Biological Field Station (Shelby County, Tenn., United States; 35°22′N, 90°1′W). We prepared tanks prior to the breeding season of anurans in early April 2009. Each tank was filled with tap water to a depth of 30.5 cm (∼613 L) and 1.0 kg of air dried leaf litter collected from a nearby deciduous forest was added. One 500-ml aliquot of concentrated plankton suspension was collected from three unused experimental tanks that were set up during August 2008. We placed a 65.58×121.92 cm piece of fiberglass composite in each tank at a 30° angle which simulated the margin of a pond. This is an important component of our design because larval *A. fowleri* do not have lungs and are negatively buoyant [31]; thus, it allowed tadpoles to position themselves in the water column without expending extra energy to respirate and provided a location for *A. fowleri* tadpoles to aggregate as observed in natural ponds. We then securely fashioned fiberglass mesh screens (1-mm mesh) as lids to each tank to prevent colonization of feral predators and competitors and to provide shading and allowed tanks to condition for 14 days before adding tadpoles. All tank preparations followed approved IACUC protocols (The University of Memphis #0650) and no further permits were necessary.

We used a 2×2×2 fully factorial design consisting of tadpoles of each species (*A. fowleri* and *H. versicolor*) reared in two pathogen treatments (*Bd* and control) for two trial durations (10 and 15 days). The resulting 8 treatment combinations were replicated 4 times and assigned randomly to the 32 experimental tanks. On 0800 on 04 May 2009 (Day 0), we brought the pathogen source and non-exposed tadpoles from the laboratory and allowed them to acclimate to the ambient temperature for 4 hours prior to placing them in the experimental tanks. For the control treatment, we placed 30 conspecific tadpoles in each experimental tank (N = 8 per species). For the *Bd* treatment, we first placed 27 non-exposed tadpoles of each species in the remaining experimental tanks (N = 8 per species). We then placed 3 *Bd* exposed tadpoles of each species in the tanks of the *Bd* treatment, equalizing the density of the two pathogen treatments.

Throughout the experiment, we placed all laboratory materials in a containment tank with bleach (6% sodium hypochlorite) to yield a 10% solution, which kills *Bd* [32]. At the completion of the experiment, we also added bleach to each tank to yield a 10% solution. The lids were then securely fashioned on the tanks and we allowed the bleach solution sit for 30 days prior to emptying the water from each tank.

### *Bd* transmission, prevalence and life history traits

On Day 10, we destructively sampled 16 experimental tanks by individually removing every larva from each tank with a small dipnet. To prevent accidental contamination of samples, we first collected all tadpoles from the control tanks. In the *Bd* tanks, we collected each larva individually, rinsed it with aged tap water, and placed it in an individual screw top vial containing MS222. Before using the dipnet again, the dipnet was thoroughly rinsed with aged tap water and briefly soaked in the mesocosm to remove any potential zoospores the previous larva deposited on the net. These methods prevented accidental transfer of *Bd* between tadpoles in, and between, each experimental tank. On Day 15, we destructively sampled the remaining 16 experimental tanks as described previously. All tadpoles were stored in 100% EtOH and brought to the laboratory where we measured the size (total length; TL) and determined the developmental stage [30] of each larva. After collecting data on size and development, we dissected the oral apparatus for quantitative PCR analysis to confirm *Bd* infection.

We used real-time quantitative polymerase chain reaction (qPCR) [33] to confirm the infection status of all tadpoles from *Bd* mesocosms and three randomly chosen control mesocosms. We also used qPCR on laboratory held tadpoles (N = 14 per species) to confirm our *Bd* exposure method. In brief, DNA was extracted from the tissue of the entire oral apparatus, which was dissected from all tadpoles immediately after collection, and stored in 100% EtOH until qPCR analyses. Each sample was run in triplicate against a *Bd* standard titration (from 10^5^ to 10^1^ zoospores) using relative qPCR on an ABI 7300 real-time PCR machine, and the pathogen treatment (*Bd* exposed or control) was unknown to the experimenter. We considered an animal as “infected” if the genome equivalent was greater than 0.1 [34].

### Behavioral Observations

During routine visits to our mesocosm array (days 5 and 10 of the experiment), we monitored our mesocosm array from 0800–1000 and made a series of observations (N = 2 per visit) on the number of tadpoles aggregating on the artificial fiberglass pond margin. Here, we define an aggregation as any number of tadpoles (≥2) that co-occurred within one-half of the width (∼30 cm) of the artificial pond margin. Thus, we did not consider tadpoles aggregating if they were co-occurred on opposite ends of the artificial pond margin. We focused on tadpole aggregations on the fiberglass pond margins because *A. fowleri* tadpoles are unlikely to aggregate on the floor of the tank because they avoid deep water (however, tadpoles sometimes positioned themselves along small ridges on the wall of the mesocosm). Because tadpoles generally aggregated on the upper one-third of the fiberglass, we considered this an effective measure of aggregation behavior because of the close proximity of tadpoles to one another on a standardized area within the mesocosm.

### Statistical Analyses

To obtain *Bd* prevalence within each mesocosm, we calculated the proportion of tadpoles infected with *Bd* at the termination of the experiment. Additionally, we obtained the zoospore equivalents of *Bd*-infected tadpoles as a measure of infection intensity. For this measure, we considered mean values per tank as the unit of analysis because measurements from infected individuals within tanks were not independent. We used analysis of variance (ANOVA) to test the main effects of species (*A. fowleri* and *H. versicolor*) and experimental duration (10 and 15 days), and their interaction, on the dependent variables proportion of tadpoles infected and their corresponding zoospore equivalents. We used square root and log transformations to normalize the prevalence and intensity data (respectively).

We measured size (TL) and stage (Gosner) per day as metrics of larval performance. We considered mean values per tank as the unit of analysis because measurements from individuals within tanks were not independent. We used multivariate analysis of variance (MANOVA) to test for the effects of independent variables species (*A. fowleri* and *H. versicolor*), pathogen treatment (*Bd* and control), and experimental duration (10 and 15 days) and their interactions on the dependent variables size and stage. Because of significant correlations between size and stage, we then used reciprocal univariate analysis of covariance (ANCOVA) on those dependent variables to control for effects of the one variable on the other.

A preliminary analysis revealed that the number of tadpoles aggregating did not differ within or between observation dates. Thus, for each pathogen treatment, we averaged the proportion of tadpoles that we observed aggregating (number of tadpoles aggregating/total number of tadpoles per mesocosm) across both observation dates. We used two-way ANOVA to test for the effects of the independent variables species (*A. fowleri* and *H. versicolor*), pathogen treatment (*Bd* and control), and their interaction on the dependent variable proportion of tadpoles aggregated. We also used regression analysis to examine the relationship between the proportion of tadpoles aggregating and the average *Bd* zoospores in each mesocosm. We used arcsine transformation for proportion data and log transformations on *Bd* infection loads to normalize our data.

All statistical analyses were performed in SPSS. Our data met the assumptions of the statistical tests used.

## Results

Infection prevalence of the subset of *A. fowleri* and *H. versicolor* tadpoles (N = 14 per species) that were held in the laboratory was 35% and 64%, respectively. This confirms our infection protocol was capable of infecting susceptible hosts. We used a conservative estimate and considered our starting *Bd* prevalence to be 10% (i.e., 3/30) and that any prevalence per tank above 10% was evidence for transmission.

One experimental mesocosm (*H. versicolor*, *Bd*, 15 day duration) developed an algal bloom that killed all the tadpoles, and was subsequently excluded from analyses. With the exception of the discarded replicate, we did not observe any appreciable mortality (<1% across all treatments). No tadpoles (N = 90) from the 3 randomly selected control mesocosms tested positive for *Bd*.

### *Bd* transmission, prevalence, and infection intensity

Although *Bd* transmission was low, we detected transmission in 4 *A. fowleri* (N = 3, Day 10; N = 1, Day 15) and 1 *H. versicolor* (Day 10) mesocosms. In addition, we found species differences in *Bd* prevalence and the average infection intensity per individual. *Bd* prevalence significantly differed between species (F_1,15_ = 15.23, p = 0.0025), where *A. fowleri* mesocosms had higher prevalence relative to *H. versicolor* mesocosms (11.7%±1.54 and 4.8%±1.60, respectively). Irrespective of species, tadpoles exposed to *Bd* for 10 days had marginally higher *Bd* prevalence relative to 15 days (F_1,15_ = 4.54, p = 0.0566); however, there was no species×duration interaction.

**Figure 1:**
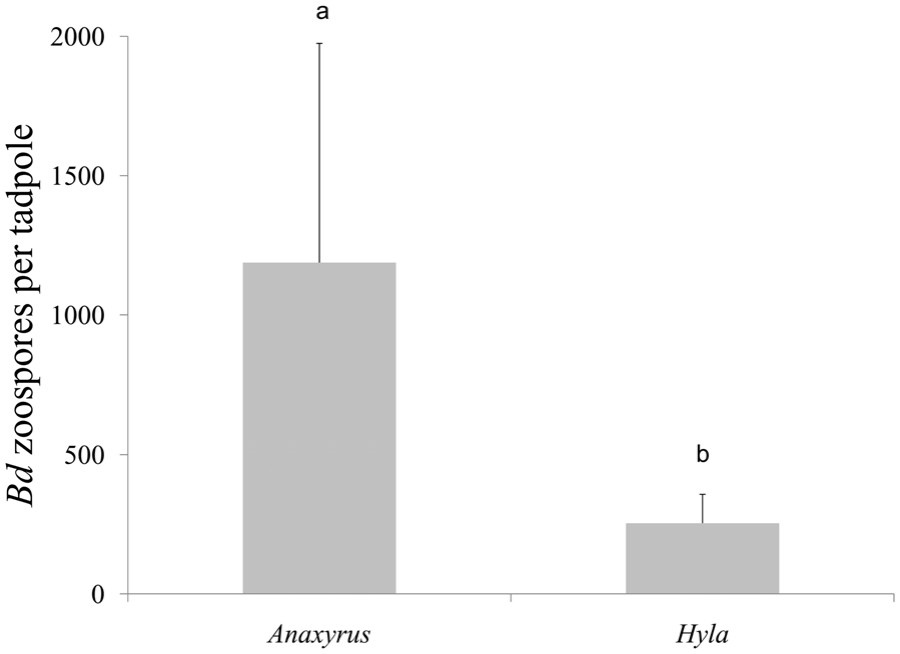
The number of *Batrachochytrium dendrobatidis* (*Bd*) zoospores detected from mouthparts of *Anaxyrus fowleri* and *Hyla versicolor* tadpoles. Different letters above histograms indicate a significant difference among treatments. The total *Bd* zoospores detected (+1 SE) on individual tadpoles within each replicate. On average, *A. fowleri* tadpoles had higher infections relative to *H. versicolor* tadpoles (F_1,13_ = 13.47, p = 0.004).

In terms of *Bd* infection intensity, *A. fowleri* tadpoles had more intense average infections relative to *H. versicolor* tadpoles (F_1,15_ = 13.47, p = 0.004; Figure 1). Additionally, we found a significant species×duration interaction, where at the 15 day trial duration, *A. fowleri* tadpoles had more intense average infections relative to *H. versicolor* tadpoles (F_1,15_ = 5.74, p = 0.035).

### Behavioral Observations

**Figure 2:**
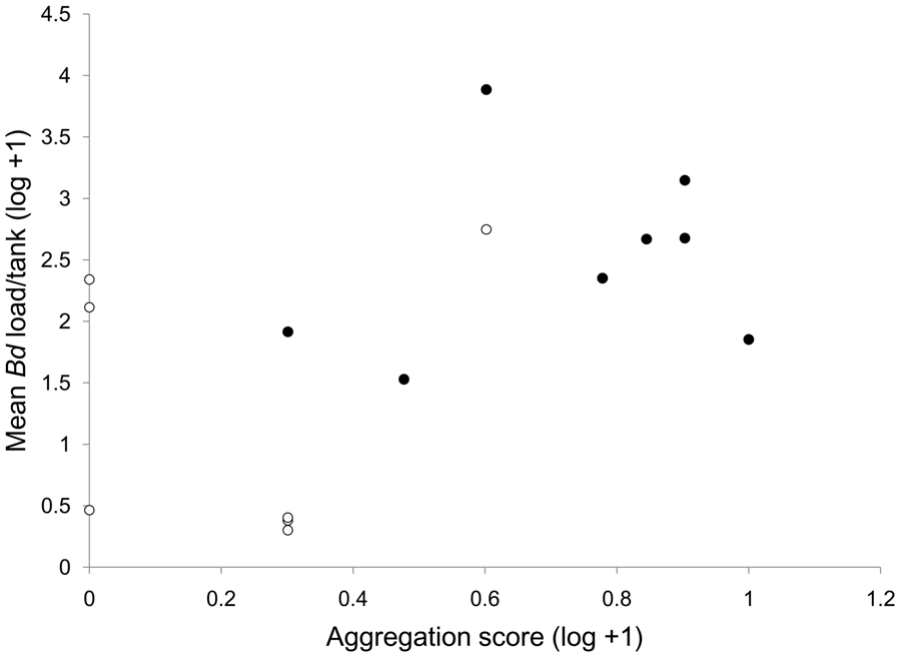
Relationship between mean *Batrachochytrium dendrobatidis* (*Bd*) load and proportion of tadpoles aggregating. *Bd* load (log+1) was averaged across all infected tadpoles and aggregation score (log+1) represents the proportion of tadpoles aggregating within a single replicate. Closed circles represent *A. fowleri* tadpoles; open circles represent *H. versicolor* tadpoles.

Since tadpole aggregations did not differ between the two observation dates (p>0.100), we used the mean proportion of tadpoles aggregating across both dates in our analysis. Neither species (F_1,32_ = 2.63, p = 0.116) nor pathogen (F = 0.149, p = 0.702) significantly affected the proportion of tadpoles aggregating on the artificial fiberglass pond margin. However, we found a significant species×pathogen interaction (F_1,32_ = 13.16, p = 0.001) on the proportion of tadpoles aggregating. Holm-Sidak post hoc analyses revealed that *A. fowleri* tadpoles aggregated significantly more (t_1,15_ = 2.84, p = 0.008) in *Bd* mesocosms relative to control mesocosms whereas *H. versicolor* tadpoles aggregated significantly less (t_1,15_ = 2.29, p = 0.030) in *Bd* mesocosms relative to control mesocosms. In addition, we found that the proportion of tadpoles aggregating was a significant predictor of *Bd* infection intensity. The total number of *Bd* zoospores detected (calculated as a sum of zoospores among infected individuals in the same replicate) increased linearly as a function of proportion of tadpoles aggregating (F_1,15_ = 4.74, p = 0.0485; Figure 2).

### Life History Traits

**Figure 3:**
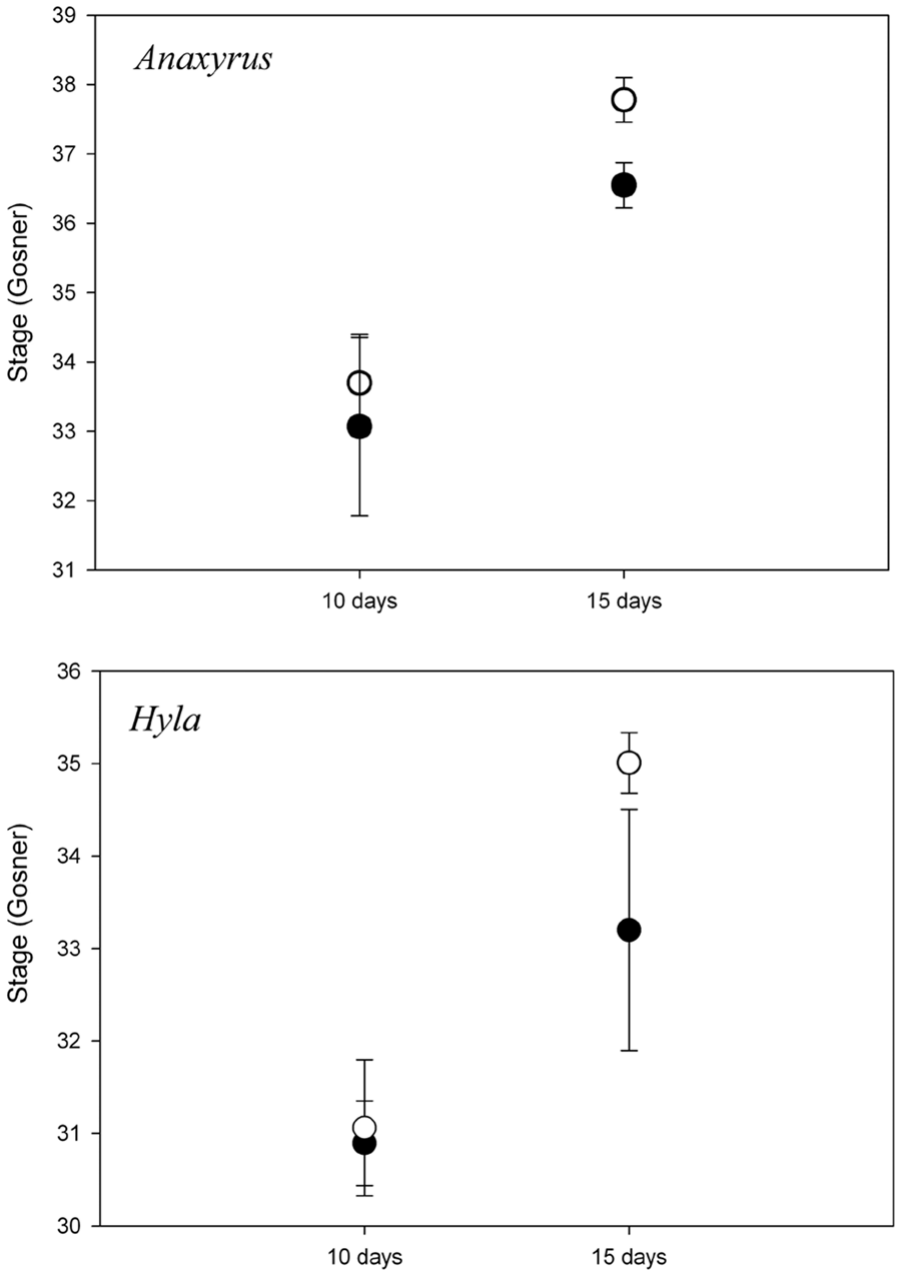
Developmental stage (Gosner) of *Anaxyrus fowleri* and *Hyla versicolor* tadpoles raised for 10 and 15 days. Data points represent tank means (±1 SE). Open circles are tadpoles raised in the absence of disease; filled circles are tadpoles raised in the presence of *Batrachochytrium dendrobatidis* (*Bd*). Overall, tadpoles raised in the presence of *Bd* were less developed compared to tadpoles raised without *Bd* (F_1,22_ = 12.57, p = 0.002).

**Figure 4:**
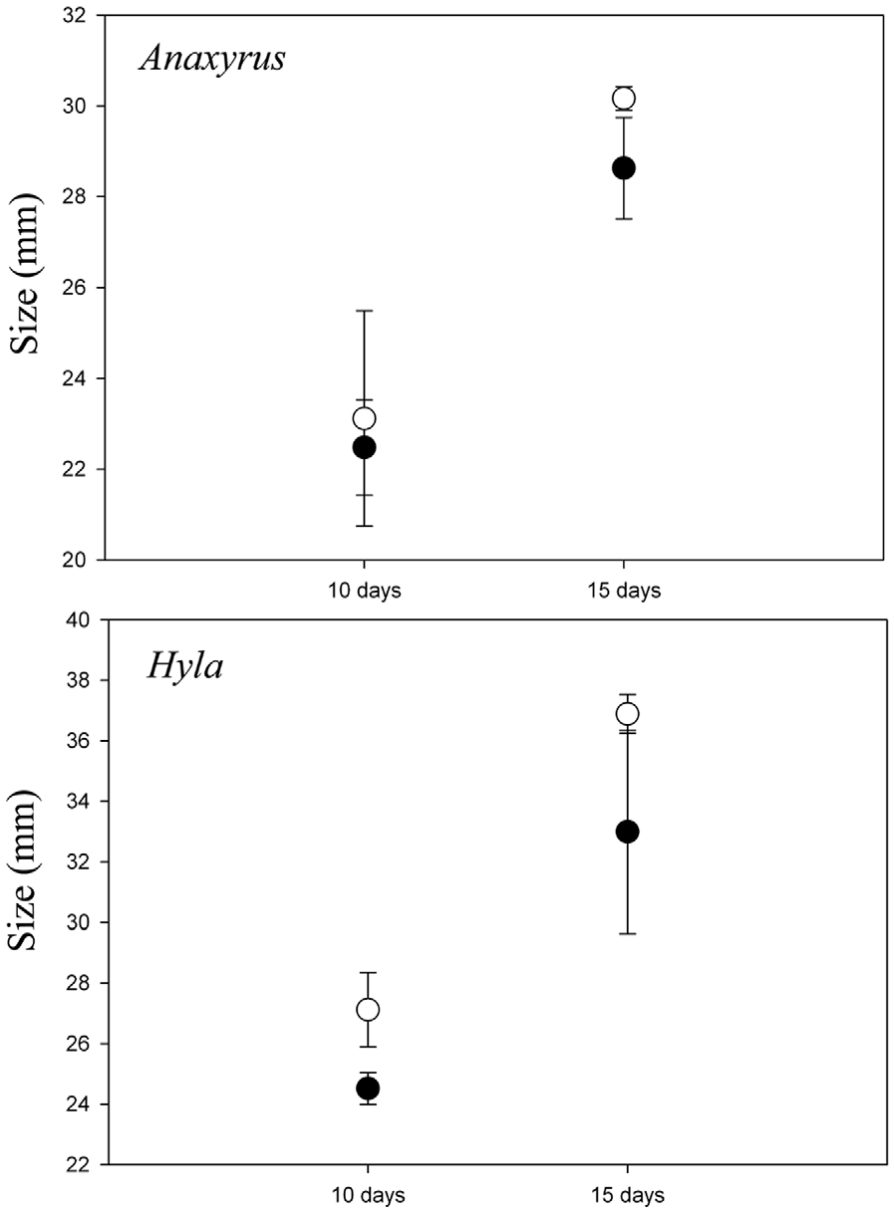
Size (mm) of *Anaxyrus fowleri* and *Hyla versicolor* tadpoles raised for 10 and 15 days. Data points represent tank means (±1 SE). Open circles are tadpoles raised in the absence of disease; filled circles are tadpoles raised in the presence of *Batrachochytrium dendrobatidis* (*Bd*). Overall, tadpoles raised in the presence of *Bd* were smaller compared to tadpoles raised without *Bd* (F_1,22_ = 8.65, p = 0.008).

There were significant MANOVA effects of species, pathogen treatment, duration, and species×duration interaction on the combined larval responses (Table 1). ANCOVA revealed significant effects of *Bd* on the size and stage of tadpoles developing in pathogen tanks. Under disease conditions, tadpoles of both species were smaller and less developed than tadpoles reared in disease-free conditions, irrespective of the experiment duration (Table 1, Figure 3, Figure 4). Additionally, at the 15 day trial duration, tadpoles of both species reared in disease conditions were significantly smaller than tadpoles in disease-free conditions for the same duration of time (Figure 4).

**Table 1:** Summary of the MANOVA and univariate ANCOVAs for larval stage (Gosner) and size (total length) for single species mesocosms of *Anaxyrus fowleri* and *Hyla versicolor* tadpoles reared in the presence of absence of *Batrachochytrium dendrobatidis* infected tadpoles at two trial durations.

MANOVA: stage, size			
Source	Wilks' λ	F	p
Species	0.032	330.57	<0.001
Pathogen	0.629	6.490	0.006
Duration	0.294	26.47	<0.001
Species×pathogen	0.987	0.149	0.863
Species×duration	0.472	12.30	<0.001
Pathogen×duration	0.775	3.248	0.058
Species×pathogen×duration	0.939	0.720	0.498

ANCOVA: stage			
Source		F	p
Species		377.26	<0.001
Pathogen		12.57	0.002
Duration		0.08	0.781
Species×pathogen		0.29	0.596
Species×duration		21.89	<0.001
Pathogen×duration		2.10	0.161
Species×pathogen×duration		0.42	0.522
Size (covariate)		146.22	<0.001

ANCOVA: size			
Source		F	p
Species		265.59	<0.001
Pathogen		8.65	0.008
Duration		1.44	0.244
Species×pathogen		0.29	0.599
Species×duration		23.70	<0.001
Pathogen×duration		4.47	0.046
Species×pathogen×duration		0.94	0.342
Stage (covariate)		172.19	<0.001

Both independent variables were used as reciprocal covariates in ANCOVAs. Significance levels for univariate tests were interpreted at 0.05.

## Discussion

Quantifying patterns and outcomes of host-pathogen interactions are essential for understanding not only the ecological implications of pathogens on their hosts, but also on ecosystem processes such as food web dynamics [35] and community interactions [36]. Determining host specific rates of transmission and infection are key challenges of host-pathogen ecology because differential transmission can significantly impact disease dynamics. Although transmission was low in our experiment, we observed intraspecific transmission (as measured through increases in prevalence above the baseline starting prevalence) in 4/8 *A. fowleri* mesocosms and 1/7 *H. versicolor* mesocosms. We also found that *Bd* prevalence marginally increased relative to the starting prevalence in *A. fowleri* mesocosms, whereas *Bd* prevalence in *H. versicolor* mesocosms decreased by the end of the experiment. When assessing transmission in our experiment, we assumed that all of the 3 pathogen source tadpoles used in each mesocosm were infected and used a conservative estimate of 10% as the starting prevalence. However, *Bd* prevalence among the subset of *A. fowleri* and *H. versicolor* tadpoles held in the laboratory to confirm our infection protocol was 35% and 64%, respectively. This suggests that not all of the 3 pathogen source tadpoles were actually *Bd*+ and that our starting prevalence might have been <10% in some of the mesocosms. If so, the transmission and prevalence that we report here might be an underestimate of actual transmission in our mesocosms.

In addition, infected *A. fowleri* tadpoles carried approximately 4× heavier *Bd* loads relative to *H. versicolor* tadpoles. Given that our data on *Bd* transmission and prevalence within *A. fowleri* mesocosms were relatively low, one plausible explanation is that our data reflect that *A. fowleri* tadpoles are generally more susceptibility to *Bd*. While this claim has some support in that other bufonid tadpoles appear more susceptible to *Bd* infection and exposure (e.g. mortality, disorientation, and lethargy; [18]), our field and laboratory data do not fully support this explanation. If *A. fowleri* are more susceptible to *Bd* relative to *H. versicolor*, we would have expected to observe a similar pattern of *Bd* prevalence in the *A. fowleri* tadpoles housed individually in the laboratory to confirm our infection protocol; however, of this subset, approximately 2× more *H. versicolor* tadpoles tested *Bd* ^+^ than *A. fowleri* tadpoles. While *A. fowleri* tadpoles might be more susceptible to *Bd*, it appears that increased *Bd* transmission and prevalence might also be influenced by other factors, such as increased social behaviors.

One possible alternative is that species differences in aggregation behavior may have increased *Bd* transmission and prevalence. Compared to disease-free conditions, we observed significantly more *A. fowleri* tadpoles aggregating when raised in the presence of *Bd* but observed the opposite effect among *H. versicolor* tadpoles. These results support our data on increased *Bd* transmission and prevalence among *A. fowleri* tadpoles and suggest that pathogen-induced behavioral changes may lead to higher pathogen transmission (e.g. [26]). A recent study found that *Bd* ^+^ *A. boreas* tadpoles (another highly social species of bufonid) associated with *Bd* ^+^ conspecifics significantly more than towards *Bd* ^−^ conspecifics [27], which suggests that *Bd* ^−^ *A. fowleri* tadpoles do not avoid *Bd* ^+^ tadpoles and that aggregations of *A. fowleri* tadpoles would increase intraspecific transmission. We also found that irrespective of species, mesocosms the degree to which tadpoles aggregated significantly predicted *Bd* infection intensity, further supporting the idea that aggregations can increase transmission and disease risk. Thus, we suggest that (a) social behaviors, such as aggregation, observed in many species of bufonid tadpoles might increase their chances of encountering aquatic pathogens and that (b) low pathogen resistance increases their infection intensity. Without an understanding of other components of the tadpole response to *Bd* (e.g., physiology and immunology), we cannot claim that differences in aggregation behavior are the exclusive factor that influenced differences in *Bd* transmission and prevalence because differences in the physiological/immunological makeup of *A. fowleri* and *H. versicolor* tadpoles may have contributed to the species differences we observed.

The duration of exposure to a pathogen can increase the probability of infection (e.g., [37]) and/or increase the duration the hosts' immune response, each of which can reduce host growth rates by reallocating energy originally devoted to somatic maintenance and development to immune function [38]. Our results also demonstrate that tadpoles reared in the presence of *Bd* have values of life history traits that imply reduced fitness. Overall, *A. fowleri* and *H. versicolor* tadpoles reared under disease conditions were less developed than tadpoles within disease-free conditions. Specifically, we found an interactive effect of trial duration and pathogen treatment on tadpole size. Compared to 10 day trial duration, tadpoles reared in disease conditions for 15 days were significantly smaller than tadpoles reared in the absence of disease. These results suggest two alternative hypotheses regarding the effects of *Bd*. First, zoospore density at the individual and mesocosm levels was not high enough to affect growth rates until after 10 days, resulting in increased pathogen effects at the longer trial duration. Alternatively, *Bd* was transmitted quickly between pathogen source and susceptible tadpoles, increasing the duration of time that tadpoles were exposed to *Bd*, thereby increasing the effects of *Bd*. Because developmental rates become fixed during late stages in ontogeny [39], [40], undersized individuals may not be able to “catch-up” in their developmental trajectory [41] after early *Bd* exposure.

Given the strong effects of pathogen treatments on larval life history traits that we measured, the low prevalence of among tadpoles of *Bd*-exposed mesocosms was unexpected. Thus, our data suggest that exposure to *Bd* is sufficient to cause reductions in growth and developmental rates, which could be due to hosts preventing, or clearing, *Bd* infections—both of which will require tradeoffs between *Bd* resistance and stage/size when tadpoles exposed to low doses of *Bd*. First, low intensity *Bd*-infections may be successfully cleared by the host shortly after infection. Although no immunopathologies have been reported for *Bd*, recent experimental evidence suggests that tadpoles exposed to a low dose of *Bd* exhibited reduced size although only 40% of the tadpoles were infected with *Bd* [24]. Second, *Bd*-infection is prevented at low doses but energy is reallocated from growth and development to prevention. Although the exact mode of *Bd* infection of anuran tadpoles is unknown, other chytridiomycete fungi attach to host specific cells prior to entering the host [42], [43]. Specific host responses, such as the activation of lymphocytes and antibodies of the larval immune system [44] may prevent *Bd* infection. Whether infection is cleared or prevented, host responses to *Bd* are likely traded off against larval growth and development. Our data, along with [24] emphasize the necessity of testing for effects of *Bd* exposure in the absence of infection.

Interestingly, we found reduced growth and developmental rates of tadpoles reared in disease conditions in the absence of severe mouthpart deformations. Two recent experiments have proposed that *Bd*-induced damage to the keratinized mouthparts of tadpoles reduce their growth and developmental rates by altering the feeding kinematics [20] and decreases their feeding efficiency [19]. During feeding, the keratinized rows of labial teeth and jaw sheaths of tadpoles and are essential for effective feeding [45]. We examined the mouthparts of each tadpole reared in disease conditions and found a low incidence of mouthpart deformation (<5%; unpublished data). The low incidence of *Bd*-induced mouthpart deformation is not surprising, given the length of our experiments. Larval *Rana muscosa* infected with *Bd* begin to lose keratin 49 days post-infection and have no keratinized jaw sheaths 147 days post-infection [46], which is much longer than maximum duration of time tadpoles from our longest trial were infected with *Bd* (i.e., 15 days). In the absence of severe mouthpart deformations, we observed strong pathogen effects on larval life history traits, suggesting that *Bd*-induced structural damage is not the only mechanism behind reduced growth and development in tadpoles.
